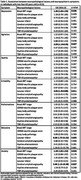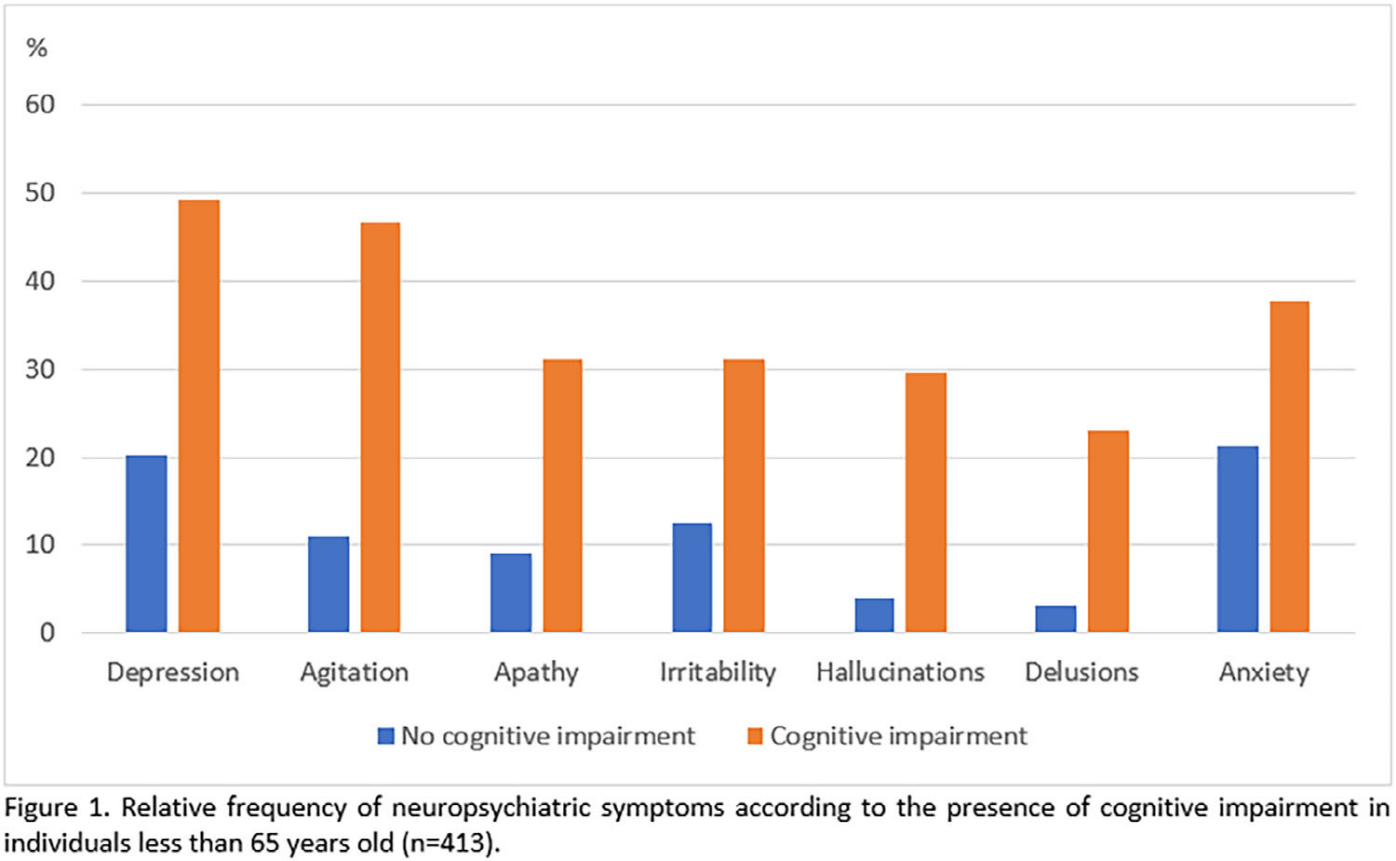# Investigating The Relationship Between Neuropathological Lesions And Neuropsychiatric Symptoms In Young And Middle‐aged Adults

**DOI:** 10.1002/alz70855_107358

**Published:** 2025-12-25

**Authors:** Renata Elaine Paraizo Leite, Lea T. Grinberg, Vitor Ribeiro Paes, Alberto Fernando Oliveira Justo, Roberta Rodriguez, Renata Eloah de Lucena Ferretti‐Rebustini, Eduardo Ferriolli, Carlos Augusto Pasqualucci, Ricardo Nitrini, Claudia Kimie Suemoto

**Affiliations:** ^1^ University of Sao Paulo Medical School, São Paulo, Brazil; ^2^ Memory and Aging Center, UCSF Weill Institute for Neurosciences, University of California, San Francisco, San Francisco, CA, USA

## Abstract

**Background:**

Neuropsychiatric symptoms (NPS) are common in neurodegenerative disorders and may precede cognitive decline over several years. Investigating the relationship between neuropathological lesions and NPS in young and middle‐aged adults can provide critical insights into the biological underpinnings of NPS and their potential as early markers of neurodegenerative disease.

**Method:**

This population‐based post‐mortem study analyzed neuropathological data from individuals aged 30–64 years, including assessments for neurofibrillary tangles (NFT), amyloid‐β burden, Lewy body (LB) pathology, TDP‐43, lacunar infarcts, cerebral amyloid angiopathy (CAA), and hyaline atherosclerosis. Post‐mortem interviews provided information on NPS using the Neuropsychiatric Inventory and cognitive status using the Cognitive Dementia Rating (CDR). We evaluated the relationship between neuropathological substrates and seven common neuropsychiatric symptoms (depression, agitation, apathy, irritability, hallucinations, delusions, anxiety) using models adjusted for sociodemographic and clinical variables, and investigated the frequency of NPS in individuals with and without cognitive impairment.

**Result:**

In 413 cases (mean age 56.7±6.1yo, 36.3% women, 60.3% white), NPS were more prevalent in individuals with cognitive impairment than in those without (Figure 1). Depression and anxiety were associated with Braak NFT stages, anxiety was associated with cerebral amyloid angiopathy, while agitation, irritability, and delusions were associated with the presence of lacunar infarcts (Table 1).

**Conclusion:**

Our findings highlight the relationship between vascular lesions and neuropsychiatric symptoms (NPS).